# Safety concerns and hidden agenda behind HPV vaccines: another generation of drug-dependent society?

**DOI:** 10.1186/s40169-016-0126-1

**Published:** 2016-12-05

**Authors:** Mahin Khatami

**Affiliations:** NCI/NIH, Bethesda, MD USA

**Keywords:** American health status, Autophagy, Baby Boomer generation, Big pharma, Bioenergetics, Cancer/medical establishment, Cancer molecular tsunami, Common sense, Fraud in cancer research, GMOs, Gut-microbiota hazard/benefit ratio, Government Welfare Program, HPV vaccines, Humanity, Immunity, ‘Medical/scientific ponzi schemes’, Mitophagy, Molecular false flags, National Cancer Institute (NCI), Obamacare insurance, Precision or Personalized medicine, Targeted therapy, The National Institutes of Health (NIH), VP Biden Moonshot Initiative, Yin-Yang of inflammation

## Abstract

Analyses of data and hidden agenda behind repeated failed outcomes of cancer research and therapy, status of American health, safety concerns for HPV vaccines and future research considerations are summarized in this commentary. A closer look at cancer science reveals that highly power structure (system) in medical establishment vs. anti-system and chaos in cancer research (‘medical/scientific ponzi schemes’) is potent recipe for failed therapeutics that kills patients but generates huge corporate profit. American health status ranks last among other developed nations despite the highest amount that USA invests in healthcare. This is a wake-up call to make sure that the evil part of human being does not prevent the health services that the public deserves. Otherwise, ‘*it does not matter how many resources you have, if you don’t know, or don’t want to know, how to use them, they will never be enough*’. Answer to cancer and improved public health is possible only by switching the current corruptive and abusive culture of ‘who you know’ to a culture of ‘what you know’. Policy makers and professionals in decision making roles are urged to return to common sense and logics that our Forefathers used to serve the public.

## Matrix of power in cancer establishment: creation of cancer-stricken society-chaos in research and therapy for huge profit

**‘** *Those who have the privilege to know, have the duty to act.* **’** Albert Einstein

Formation of a highly ordered and sophisticated medical hierarchy (establishment) in the nineteenth/twentieth century within higher education institutions (e.g., medical schools, organizations) was supported by businessmen and philanthropists with motives to profit from the sale of drugs (reviewed in 1). The power of establishment grew since 1955 when public was intentionally inoculated with million doses of virus-contaminated polio vaccines, which sharply increased the deadly cancer incidence in the current ‘baby boomers’ generation, particularly in America. In addition to increased cancer incidence and mortality, numerous other disabling acute or chronic illnesses [e.g., poliomyelitis, vasculitis, autoimmune and neurodegenerative diseases or vaccine-associated paralytic polio (VAPP)] are reported as the results of public vaccination with virus-contaminated polio vaccines that made American health status at the bottom of other healthy nations [1–3].^1^^,^ 2 ^,^ 3 ^,^ 4 ^,^ 5 The abusive power of establishment intensified since 1971, when the Cancer Act, signed by President Nixon, increased cancer research funding of National Cancer Institute (NCI)/NIH to 1.6 B, so that cancer problem be solved in 8 years! The establishment has been successful in collecting/spending several trillions of dollars from public and private resources ($1.6 trillion spent in 2008 alone on research, drug development, clinical trials and care) with claims of ‘targeted’ therapy, ‘precision’ or ‘personalized’ medicine, including the recent failed attempts for ‘immunotherapy’ [1].

In addition to surgery, current treatments options (chemotherapies) primarily use potent apoptotic factors, specific growth factor inhibitors (monoclonal antibodies), stem cell transfer, or inhibit check point proteins of T cells or genetic mutations of PDs in monocytes and claims of immunotherapy [1]. Treatments are often combined with partial or total body irradiation (radiotherapy). These clinical approaches induce ‘immune tsunami’ or ‘cytokine storm’ in an already immune compromised body of patients and destroy integrity and function of vital organs such as the liver, kidneys, bone, muscle and vasculature resulting in life-threatening side effects [e.g., drug-resistant and relapse, cachexia, sarcopenia, fatigue, thromboembolism and multiple organ failure (MOF)] and loss of lives [1, 4–8]. Such highly toxic treatments resemble the severe reactions that are described for potent pathogen-induced acute inflammatory diseases and rapid generation of cytokine storm in such diseases as sepsis, meningitis, salmonella poisoning, pneumonia or major trauma often leading to MOF or death [1, 4, 5].

Therefore, there is no surprise that outcomes of such illogical approaches (‘medical/scientific ponzi schemes’) have failure rates of 90% (±5) for solid tumors [1, 4, 5].

War on cancer is a very expensive Government Welfare Program for members of the establishment and their surrogates who enjoy career longevities of 40–65 years and who are entitled to continuously receive large sums of travel funds and grants with little/no review processes or producing anything of value to benefit the society [1].^6^ In 2013, American Association for Cancer Research (AACR, strong lobbying group, established in 1907) shamelessly boasted that 1/3 (33%) of all women, and 1/2 (50%) of all men develop cancer in their lives and that they need more money to ‘Stand Up To Cancer’!

The establishment is entitled to glamorize and publicize too many drugs or vaccines with little/no ethical or safety considerations for short-, or long-term health hazards of such projects. Policy makers in Congress have no clues how to assess worthy or worthless projects as they depend on advice of members of establishment and their surrogates who occupy high positions and scientific recognitions, including Nobel prizes, as the only ‘authorities’ to defend such illogical projects that are more like ‘building too many expensive bridges to nowhere’; and identifying ‘molecular false flags’ based on false foundations [1, 5].^7^ The establishment tolerates no challenge or objection from competent and independent scientists. Independent professional views are perceived as ‘threat’ to the establishment and professionals become subjected to heavy harassment, bullying, unethical and criminal practices of retaliation and elimination [1].

With the availability of modern technologies, decision makers in the government, academia or Big pharma become narrowly experts in their fields of ‘omics’ (e.g., genomic, proteomic, lipidomic, glycomic, metabolomic) and know details of structures and substructures of viruses, bacteria, parasites (microbiotics), carcinogens and endless broken/defective molecules (e.g., somatic mutations of growth or apoptotic factors, enzymes, receptors) or how to inhibit them in experimental models of tumors or clinical trials [1, 4, 5]. However, cancer remains an imaginary problem (‘it is too many diseases’) to solve. Public deception on cancer science reminds us of the statement of Philip Zelikow ‘*The creation and maintenance of public myths exert a powerful influence*’ [1].^8^

**Table:** 

Lack of oversight and accountability and abuse of funds on too many failed projects made cancer research a myth making machine by ‘intellectuals’ who portray cancer as too difficult a problem to solve!	

**Fig. 1:**
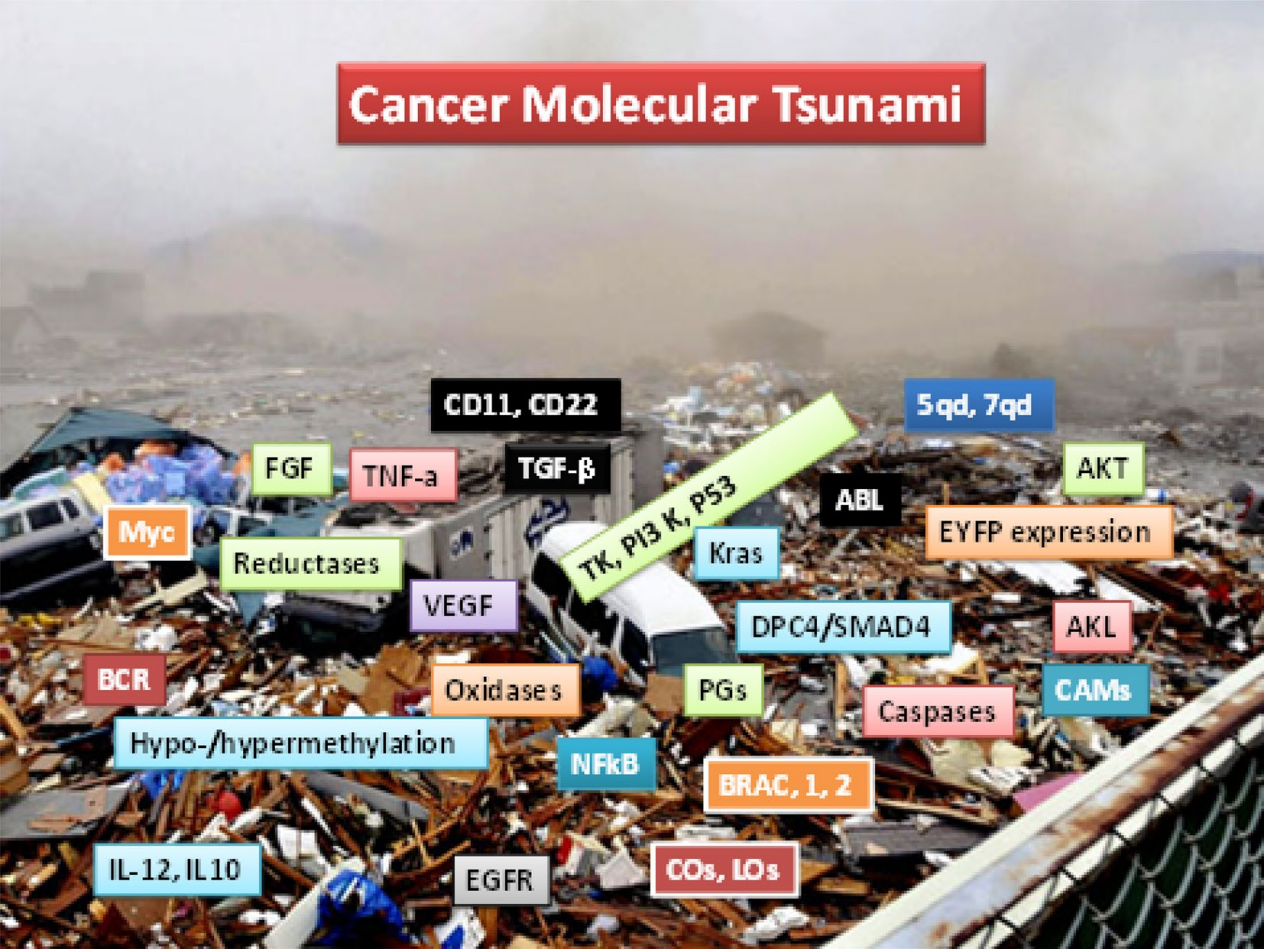
‘Cancer Molecular Tsunami’. Photo (Japanese Tsunami, 2011) resembles severely disrupted integrities of molecular, cellular and sub-cellular structures of immune and non-immune systems in site-specific cancers. Candidate drugs are based on endless identification of defective molecules, genetic mutations of over-, under-, or co-expression of growth and apoptotic factors, cell surface and receptor molecules, decoy receptors, cytokines/chemokines, enzymes/proteins or vascular and membrane components (e.g., p53, ALK, AKT, NFkB, PI3K, MAPKs, Myc, Hsp-90, VEGF, EGFR, IGF, FGF, IFN-γ, TNFdR, MMPs, CRP, S1P, CD44, CD73, CD146, CD166, CD90, CD105/CD1-5, PTEN, TGF-β, PDL-1, IL-12, COs, LOs, TLRs, mTOR, caspases, tryptase, chymase, oxidases, PGE2). Photo Source: The Internet. Reproduced from Ref [1]; all rights reserved

With hundreds of thousands of disturbances in network of molecular, neuronal, immunological, vascular, metabolic, bioenergetics, physical and mechanical properties that are present in cancer molecular tsunami, who could ever claim that inhibiting one or two or 10 molecules would correct or treat any solid tumor? (Fig. 1) [1, 4, 5, 8–10].^9^^,^ 10 ^,^ 11

These ‘specialists’ whose career longevity depends on defending such worthless projects remind us of Rumi’s spiritual statement that ‘*People cannot see the camel in the minaret but they can see the hair in its nose!*’^12^

Peyton Rous said it best that ‘*A hypothesis is best known by its fruits. What have been those of the somatic mutation hypothesis? It has resulted in no good thing as concerns the cancer problem, but in much that is bad… Most serious of all the results of the somatic mutation hypothesis has been its effect on research workers. It acts as a tranquilizer on those who believe in it.*’ This statement was made in 1959, well before genetic studies in cancer and claimed ‘targeted’ therapies were put on steroids!

[1, 4].^13^

Loss of patients lives, particularly the loss of politicians and celebrities or their families seem to be great incentives for cancer establishment and its world’s largest lobbying group to go before Congress and claim that they made ‘remarkable achievements’ but need ‘more money’ to continue! It is outrageous that even after patients lose their lives to toxicities of drugs, money is collected in lieu of ‘flowers’, or the victims leave small or large fortunes in their ‘wills’ to help ‘cancer research!’

There is a peculiar absence of systematic investigation to logically understand what triggers initial events in the loss of immunity (immune surveillance) originally described by Burnet in 1957 [1]. Except for ‘accidental’ discoveries that our research team established in 1980s on models of acute and chronic inflammation, there is little/no evidence on early stages of immune dysfunction toward multistep tumorigenesis and angiogenesis, although numerous circumstantial evidence on a role for inflammation in cancer have been documented. In 1980s we were not involved in cancer research and had no idea of the importance or significance of the findings for cancer research until I joined NCI/NIH in 1998. Analyses of data provided the first series of evidence for a direct link between inflammation and initial immune response alterations including the first report on sequential interactions and synergies between host immune and non-immune cells and those of activated recruiting inflammatory cells in the direction of tumorigenesis and angiogenesis [1, 4, 5]. We further defined effective immunity as the balance between two tightly regulated and biologically opposing arms of Yin (tumoricidal, growth-arrest) and Yang (tumorigenic, growth-promote) of acute inflammation, an amazingly successful network of biological signals from immune and non-immune systems (e.g., vasculature, neuronal, metabolic, hormonal activities) for protecting the body against all intrinsic and extrinsic elements that are perceived harmful to body’s survival throughout life [9, 10].

## Safety concerns and hidden agenda for publicizing HPV vaccines: abuse of affordable care insurance and moonshot initiative: creating another drug-dependent sick society?

On September 7, 2016, NCI presented a document “Cancer Moonshot’s Blue Ribbon Panel” to National Cancer Advisory Board. It identified 10 priorities for cancer research including HPV vaccination. The document rehashes the same fuzzy approaches that have been used in the last six decades for cancer research and therapy or vaccines with different spins [1].^14^ The document reminds us of the tactics that were used in 1970s by CDC director for urgently seeking extra fund for swine flu vaccination. Review of an interesting article “The Swine Flu Affair” [11] resembles the scenario that establishment described for targeting young population for HPV or meningitis vaccines and justifying additional funding.

A wide range of vaccine-related health problems including autism (measles vaccines), multiple sclerosis (hepatitis B), menangioencephalitis (Japanese encephalitis), Guillian-Barre syndrome and giant cell arthritis (influenza), encephalomyelitis (semple rabies), neurological problems (e.g., H1N1, swine flu) have been reported in literature. The total number of death and diseases that were caused by polio, swine flu and other specific vaccines, even BCG vaccines are greater than diseases these vaccines were intended to prevent [1, 12–14].^15^ The rush for HPV vaccination is no exception as described below.

Human papilloma viruses (HPVs) are small heterogeneous family of at least 130 different viruses (HPV types) of double-stranded DNA whose potencies and genomic structures evolve in host and are different from individual to individual, tissue to tissue and time to time. HPVs have been identified in organs/tissues (e.g., skin, larynx, vagina, penis, esophagus, conjunctiva, bronchus, paranasal sinuses, tracheo-bronchial and oral mucosa, anogenital tract, urethra) in diseases such as genital warts, recurrent respiratory papillomatosis, low-grade and high-grade squamous intraepithelial lesions (SILs) and anal, vaginal and cervical cancers [1, 15–17].^16^

Emphases on production of specific vaccines to inactivate segments of viral structures such as HPVs DNA structures or expression products, while not effective to prevent specific diseases (e.g., cervical cancers), long-term effects of HPV vaccines (Gardasil™, or Cervarix™) could contribute to initiation of health problems during aging, if not sooner. The genomic structures of HPVs in vaccines (e.g., inactivated high potency particles) could disturb host tissues in a variety of mechanisms (e.g., mutations of DNA components or integration into host chromosomes and instability of genomic substructures). Exposure to viral particles and adjuvant (aluminum) in vaccines, along with routine exposures to other immune disruptors are ‘antigen overload’ for immune system that could shift the induction of chronic health problems (e.g., increased asthma, ocular or skin allergies, hot flashes, gastrointestinal conditions or neurological and autoimmune diseases) that are features of aging to younger individuals [1, 18, 19].

Professionals and policy makers in other countries started raising serious questions about the “scientific uncertainties related to the safety of HPV vaccines…Sloppy science, combined with unprofessional and unfair criticism of independent research, such as the one the EMA raised against the diligent Danish researchers, is a serious threat to scientific progress and public health…”.^17^ Recent clinical data already suggest adverse effects of HPV vaccines, composed of genotype-specific capsid proteins variations (e.g., HPV-16, HPV-6 or HPV-11) or expression of detectable HPVL1 protein and DNA fragments in aluminum-containing adjuvant, of virus-like-particles-VLPs by DNA recombinant methodologies [15, 16].^18^

We suggested that exposures to specific virus-containing vaccines, by inhibiting/inactivating specific high risks (‘potent’) segments of viral DNA lead to inflammatory conditions that would influence the homeostasis and dynamics (ecosystem) of host microorganisms (e.g., GI track, skin) in young adults. Altering hazard/benefit ratios of microbiota are important contributing factors in ‘antigen overload’ for immunity and cross reactivity of antibodies against antiviral immune complexes and induction of age-like chronic diseases in younger adults. Our observations that newborn guinea pigs born from sensitized parents showed strong allergic reactions upon 1st or 2nd challenge with antigen, suggesting genetic predisposition of fetus [1, 4, 5] are indirectly supported by clinical data [1, 18, 19].

Again, a great deal of investment have been directed to identify details of structures and substructures of microorganisms, carcinogens or expression products and mechanisms of actions of evolving numerous infective agents [e.g., HPV, polio, rous sarcoma, herpes, AIDS, EBOLA, influenza, measles, hepatitis (A, B and C), LPS, meningitis or Zika]. However, what initiates altered tissue response dynamics toward multistep diseases or cancers remains a mystery [1, 11–17].

The hidden short- and long-term agenda behind making HPV or meningitis vaccination as priority projects seem the availability of funds through Obamacare insurance and Moonshot Initiative. There should be no surprise that the cost of individual insurance keeps going up. Sixty-nine cancer centers urged HPV vaccination and thus-far, 80 million doses of HPV vaccines ($200–260/dose) consumed by healthy public [1].^19^^,^ 20

It is painful to project that the sick status of ‘baby boomers’, created half a century ago could be repeated, if not already started, by vaccinating the public with HPV or other vaccines (e.g., meningitis, shingles, flu), whether or not vaccines are contaminated with live viruses. Such fraud approaches could present grave health consequences for future generation (s), if the policy makers, professionals and public do not reflect on the fact that ‘intellectuals’ in health system who were responsible for improving public health are destroying it.

## Future considerations: use of common sense to advance cancer science and effective vaccines for healthier society

Like cancerous cells, allergen and pathogenic components in vaccines are intrinsic or extrinsic foreign entities to be neutralized and cleared or ignored by effective immunity. Microorganisms and defective cancerous cells co-exist in the highly ordered multilayered cellular organization of host as long as their numbers or potencies do not overwhelm the complex defense mechanisms, the balance in Yin and Yang of self-terminating properties of acute inflammation. However, effective immunity can be weakened or lost by frequent exposures to biological and environmental hazards, particularly during aging process. Suppressed immunity (sustained oxidative stress) provides opportunities for pathogens or cancerous cells to cause damage to host immune dynamics and initiation of health conditions such as allergies or other inflammatory diseases or cancers [1, 4, 5, 9, 10, 19].

We recently presented evidence-based interrelated hypotheses that cancer is a severe and cumulative delayed type hypersensitivity reaction in site-specific tissues [1, 19]. Histamine, at low circulating level was suggested as blueprint for maintaining oxidative stress that contributes to tissue necrosis or growth in immune-privileged or immune-responsive tissues and induction of neurological or autoimmune diseases or tissue growth promotion. Histamine, an alkaline, contributes to dysfunction of mitochondrial and ribosomal activities (mitophagy and autophagy), tissue acid–base balance and bioenergetics (e.g., adenosine, ATP/ADP/AMP, Ca^+2^, H^+^/Na^+^/K^+^ transporters and exchangers), membrane structures (e.g., receptor or surface molecules, ionic and water channels). Among key components for future understanding of the effective immunity are the crucial roles that mitochondria and autophagy play in maintenance of Yin-Yang and energy-dependent events in protein/lipid recycling and biosynthesis of structural proteins (e.g., metabolism of branched amino acids) involved in architectural integrity of tissue anchoring and contact inhibition [1, 5, 19, 20].

**Fig. 2:**
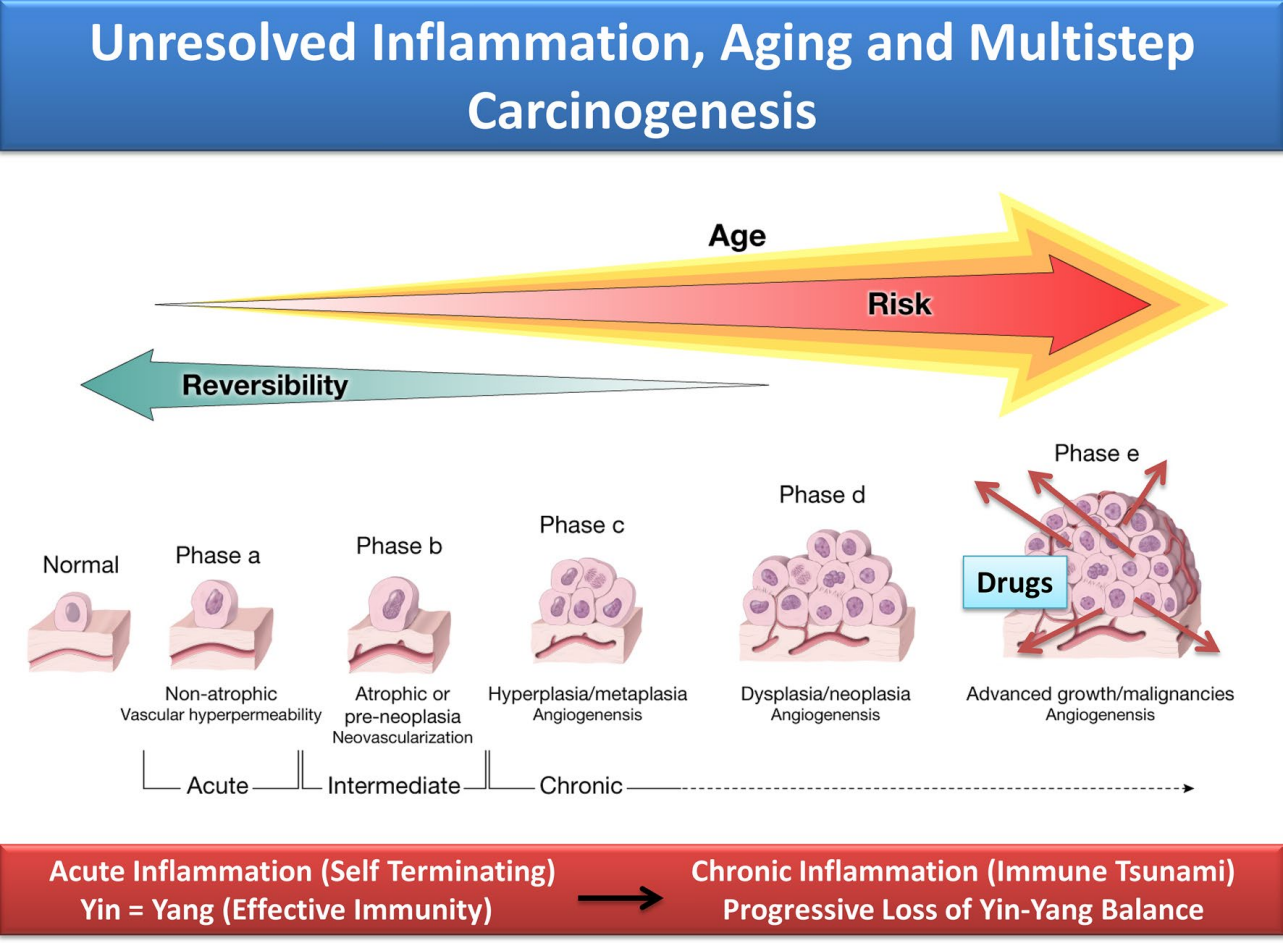
Schematic demonstration that aging and unresolved inflammation are co-risk factors in developmental phases of immune dysfunction in multistep tumorigenesis and angiogenesis. The *left panel* depicts initial stages of our ‘accidental’ discoveries on inflammation-induced identifiable immune dysfunction in ocular tissue responses during (*a*) acute phase responses or self-terminating (reversible) events; (*b*) intermediate phase, down-regulation phenomenon accompanied with mild tissue atrophy and neovascularization, potentially reversible; and (*c*) chronic phase, induction of massive lymphoid hyperplasia and tumorigenesis and angiogenesis. The *right panel* represents chronic inflammation and continued stages of tissue growth (*d*, *e*) advancing to cancer malignancies and angiogenesis in site-specific tissue. The complex scheme demonstrates that majorities of translational medicine and clinical trials are conducted in identification of endless damaged molecules at advanced stages of carcinogenesis and drug use (*red arrows* in phase *e*, ‘cancer tsunami’). Modified from, Exp Opin Biol Ther; Informa Healthcare, 2011 [10]. All Rights reserved

Designs of universal vaccines, or prophylactic candidates that would generally enhance/promote or stabilize the innate immune cells (e.g., mast, dendritic and natural killer cells or macrophages) could also influence resting capability of adaptive immune cells (e.g., T and B/plasma cell polarities) and corresponding crosstalk with non-immune systems. Pathogen-(stimuli) induced early alterations in immune dynamics are likely reversible, preventable/correctable or druggable (Fig. 2) [1, 5, 10, 19]. Logical efforts for therapies or vaccines are intellectually challenging, particularly because after investing trillions of dollars on too many worthless projects, we know very little on early molecular dynamics that alter response dynamics of site-specific tissues toward genesis of diseases. Careful designs of logical studies are anticipated to lead to identification of suitable disease markers, accurate formulation of risk assessment and cost-effective translational medicine toward a healthier society that the public deserves.

## Concluding remarks

For over a century all directors of NIH and other governmental health agencies, cancer centers and organizations, medical schools, Big Pharma and food industry (producers of genetically modified organisms/GMOs) have been physicians (with MD degrees). The only formal duty of these leaders was to improve and promote public health, prevent diseases and save American lives. However, despite excessive investment of American resources for healthcare the opposite has occurred. American health ranks last of 11 or last of 17, compared with other developed nations. Majority of vaccines that were designed to prevent diseases caused more death and diseases than public exposures to infective agents.

Policy makers and public should take a closer look at the long-lasting ‘medical/scientific ponzi schemes’ that cancer establishment created to control a drug-dependent sick society. Millions of cancer patients who enter clinical trials are treated with drugs (poisons) and procedures that postpone their death-sentence for short duration, while their resources (insurance and personal assets) are drained! In this medical ponzi scheme, not only trillions of dollars wasted on ‘molecular false flags’, but millions of precious lives were lost to such illegal, unethical and horrendous crimes against humanity.

Instead of using common sense to promote health and prevent or delay the onset of age-associated diseases, medical establishment has managed to gradually alter and destroy the natural immunity of Americans public and shift onset of diseases to younger age for increasing the population of sick people and pushing drug sale.

This is a wake-up call to make sure that the evil part of human being does not prevent the health services that the public deserves.

Answer to cancer and increased public health is possible only if policy makers and cancer-stricken public seriously realize that the *might* of establishment over the *right* of science must be drastically reversed.

Decision makers in Congress who appropriate funds and those who direct medical sciences, should return to the forgotten values of common sense and logics that our Forefathers used for serving the public. After all ‘*we may be intelligent, but if not able to think and love well being of others, we use the intelligence against humanity*’.

## Footnotes

1 History of polio—BBC News http://www.bbc.com/news/health-17045202--. By 1988, polio had disappeared from the US, UK, Australia and much of Europe but…’.

2 Kelleigh Nelson, July 29, 2010, American Citizens as Guinea Pigs (part 2): The polio vaccine contamination with SV-40.

3 Grace Rubenstein: New Health Rankings: Of 17 Nations, U.S. is Dead Last Jan 10, 2013.

4 Davis K, Stremikis K, Schoen C, Squires D: *Mirror, Mirror on the Wall, 2014 Update: How the U.S. Health Care System Compares Internationally,* The Commonwealth Fund, June 2014.

5 Al Sears MD: The greatest health scam in history used President Eisenhower as a pawn. http://www.alsearsmd.com/…/the-greatest-health-scam-in-history-used-president-e.

6 Senator Grassley letter to NIH and NCI directors, October 2010, on excessive travel expenses of individuals for a 2 year period. Senator Grassley raised concerns to the reprimand given to an NCI staff in ethics office who did her job for questioning travel expenses of NCI/NIH staff.

7 Budget Hearing-National Institutes of Health, Wednesday March 16, 2016; witnesses included Drs. FS Collins (NIH Director, testimony), D Lowy, (Acting Director, NCI) AS Fauci (NIAID Director)-with Subcommittee Chairman Rep. Tom Cole and Hal Rogers Chairman http://appropriations.house.gov/calendar/eventsingle.aspx?EventID=394449.

8 Phillip Zelikow; one of the architects behind creation of war against powerless nations.

9 Medscape, December 5, 2011 (Washington DC) reported by Dr. Fojo (NCI) 'zero [is] the number of targeted therapies that prolonged survival by one year' when compared with conventional treatment.

10 Clifton Leaf: Truth in Small Doses-Why we’re losing the war on cancer-and how to win it.

11 Butcher Lola: Unintended Consequence: How Government Policies Have Increased the Cost of Cancer Care; Part 1, Medicare Pay Change Triggered Care Migration, Oncology Times, September 10, 2014.

12 Rumi, 1207–1273, (13th Century), Iranian poet, Jurist, Islamic Scholar, Sufi Mystic. Meeting with the Dervish Shams-e Tabriz (1244) completely transformed Rumi’s life from an accomplished teacher and jurist into an ascetic. Rumi’s influence transcends national boarders and ethnic divisions.

13 Peyton Rous, Nobel Laureate in Physiology or Medicine 1966, Father of Virology.

14 Cancer Moonshot: Report of the Cancer Moonshot’s Blue Ribbon Panel to the National Cancer Advisory Board, September 7, 2016 (NCI document); also publication of NCI and Vice President Moonshot Task Force, summary of final report on five ‘strategy’ and plans for achieving the Moonshot’ Initiative, approval of President Obama ‘Architectural Framework’, White House Press Conference, October 17, and Cancer Letter, October 21, 2016.

15 Barbara Loe Fisher: The Vaccine Reaction. “CDC admits flu shots fail half the time”, “…Public health doctors push ineffective, reactive Flu vaccine”—Health Impact News; October 24, 2016.

16 “Is The U.S. Becoming a Police State to Force Mandatory Vaccination?”; and “American Academy of Pediatrics wants a Police-State approach to vaccination”, Health Impact News, September 14, 2016.

17 “Complaint to the European ombudsman over maladministration at the European Medicines Agency (EMA) in relation to the safety of the HPV vaccines”. Letter signed by professionals regarding HPV safety concerns; Nordic Cochrane Centre, Copenhagen, Denmark. October 10, 2016. Link to http://nordic.cochrane.org/sites/nordic.cochrane.org/files/uploads/ResearchHighlights/Complaint-to-ombudsman-over-EMA.pdf.

18 Norma Erickson SaneVax Inc. Dr. Sin Hang Lee recommends China postpone HPV vaccinations. Health Impact News, accessed October 31, 2016. https://healthimpactnews.com/2016/dr-sin-hang-lee-recommends-china-postpone-hpv-vaccinations/.

19 The Cancer Letter, ‘Sixty-Nine Cancer Centers Urge HPV Vaccination’; January 29, 2016.

20 Clair Dwoskin: “Allergens in vaccines are causing life-threatening food allergies”; also 80 million doses of HPV vaccines consumed by public, Health Impact News, accessed October 30, 2016.
